# Identification of Key Genes in Diabetic Retinopathy Using Bioinformatics and Machine Learning Approaches

**DOI:** 10.7759/cureus.99936

**Published:** 2025-12-23

**Authors:** Xiaoyan Zhu, Lulu Lian

**Affiliations:** 1 Department of Ophthalmology, Gansu Provincial Maternal and Child Health Hospital, Lanzhou, CHN

**Keywords:** bioinformatics, col6a2, diabetic retinopathy, extracellular matrix, machine learning

## Abstract

Objective

This study aimed to identify key genes associated with diabetic retinopathy (DR) by applying bioinformatics and machine learning techniques to publicly available transcriptomic datasets. We further evaluated their diagnostic performance and explored their potential biological functions and upstream regulatory mechanisms, providing a theoretical basis for the early diagnosis and molecular-targeted therapy of DR.

Methods

DR-related transcriptomic datasets GSE94019 and GSE60436 were obtained from the Gene Expression Omnibus (GEO) database, with GSE94019 serving as the training set and GSE60436 as the validation set. The data were then subjected to normalization and differential expression analysis. Feature genes were selected using the Least Absolute Shrinkage and Selection Operator (LASSO) regression and Support Vector Machine-Recursive Feature Elimination (SVM-RFE) algorithms. Overlapping genes were identified as key candidates. Diagnostic performance was evaluated by plotting receiver operating characteristic (ROC) curves using the R package pROC. Functional enrichment analysis, including Gene Ontology (GO) and Kyoto Encyclopedia of Genes and Genomes (KEGG) analyses, was performed on differentially expressed genes (DEGs) associated with the key gene. Potential upstream miRNAs and lncRNAs were predicted using the miRanda, miRDB, TargetScan, and spongeScan databases, and a lncRNA-miRNA-mRNA regulatory network was constructed.

Results

A total of 790 DEGs were identified, including 370 upregulated and 419 downregulated genes. Cross-validation using LASSO and SVM-RFE identified Collagen Type VI Alpha 2 Chain (COL6A2) and LINC01247 as key genes. COL6A2 was significantly upregulated in the DR group. ROC analysis revealed high diagnostic accuracy, with area under the curve (AUC) values of 1.00 (training set) and 0.89 (validation set). In contrast, LINC01247 was significantly downregulated, but its AUC values were 1.00 (training set) and 0.52 (validation set), indicating limited diagnostic value; thus, it was excluded from further analysis. Functional enrichment centered on COL6A2 suggested that its associated DEGs were involved in aberrant extracellular matrix (ECM) organization, cell adhesion, angiogenesis, and inflammatory responses. Moreover, regulatory network analysis indicated that hsa-miR-762 and hsa-miR-29a-3p may indirectly regulate COL6A2 expression by competitively binding multiple lncRNAs (e.g., PABPC1L2B-AS1 and RP11-223P11.3), forming a potential ceRNA regulatory axis.

Conclusion

This study identifies COL6A2 as a key gene in DR, characterized by significant upregulation in DR tissues and close involvement in ECM remodeling, cell adhesion, and angiogenesis. These findings provide novel molecular targets and theoretical insights for elucidating the molecular mechanisms of DR and for improving early diagnostic strategies.

## Introduction

Diabetic retinopathy (DR) is one of the most common and severe microvascular complications of diabetes, and a leading cause of blindness among adults worldwide [1]. According to statistics from the World Health Organization and the International Diabetes Federation, the number of individuals with diabetes is rapidly increasing, and is projected to exceed 700 million by 2045, with approximately one-third expected to develop varying degrees of retinopathy. The onset and progression of DR are closely associated with endothelial dysfunction, chronic inflammation, oxidative stress, and aberrant extracellular matrix (ECM) remodeling induced by prolonged hyperglycemia [2]. Although current clinical interventions - such as laser photocoagulation, anti-vascular endothelial growth factor (VEGF) therapy, and vitrectomy - can delay disease progression, early diagnosis remains a major challenge, and reliable molecular biomarkers are still lacking. Therefore, elucidating the molecular mechanisms of DR and identifying key genes is of great scientific and clinical significance.

With the rapid development of high-throughput sequencing technologies, the availability of large-scale transcriptomic datasets has created new opportunities to investigate the molecular basis of complex diseases. Bioinformatics approaches allow the integration of multi-omics data to systematically identify disease-associated genes, signaling pathways, and regulatory networks, thereby uncovering potential pathogenic mechanisms. In recent years, bioinformatics analyses based on public databases, such as Gene Expression Omnibus (GEO), have become an important research avenue in DR. Previous studies have shown that inflammatory responses, apoptosis, angiogenesis, and abnormal ECM metabolism play pivotal roles in DR, and genes such as VEGFA, ICAM1, and MMP9 have been implicated in its pathogenesis and progression [3]. However, most existing studies rely primarily on traditional differential expression analyses, which may be influenced by data noise and subjective filtering criteria, making it difficult to accurately pinpoint the most diagnostically or prognostically valuable key genes.

In recent years, machine learning techniques have developed rapidly in the field of bioinformatics, offering new strategies for biomarker discovery and predictive model construction in complex diseases. Machine learning enables algorithmic models to learn feature patterns from high-dimensional data, thereby facilitating more efficient identification of potential key genes and improving model performance. Among these methods, the Least Absolute Shrinkage and Selection Operator (LASSO) regression [4] and Support Vector Machine-Recursive Feature Elimination (SVM-RFE) algorithms [5] are widely used, due to their efficiency and stability in feature selection. Combining these two methods can reduce overfitting while improving the reliability and robustness of the identified candidate genes.

Against this background, the present study aimed to identify key genes closely associated with DR by integrating bioinformatics and machine learning approaches, using DR-related transcriptomic datasets retrieved from the GEO database. First, the dataset was normalized and subjected to differential expression analysis. LASSO regression and SVM-RFE were then applied independently to select feature genes, and their intersection was used to determine robust key genes. Subsequently, the expression patterns and diagnostic value of the identified gene(s) in DR were evaluated. Furthermore, functional enrichment analyses were performed to explore their potential roles in biological processes (BPs) and signaling pathways. Finally, upstream lncRNA-miRNA-mRNA regulatory networks were constructed, using public databases to investigate possible post-transcriptional regulatory mechanisms.

## Materials and methods

Data acquisition and preprocessing

The transcriptomic datasets GSE94019 [6] and GSE60436 [7], both related to DR, were retrieved from the GEO database. The dataset GSE94019 served as the training set, comprising four normal samples and nine DR samples. It was analyzed using differential expression analysis, LASSO regression, SVM‑RFE, ROC curve analysis, and enrichment analysis. The dataset GSE60436 was used as the independent validation set, which included three normal samples and six DR samples, and was subjected to ROC curve analysis to evaluate the diagnostic performance of the key genes identified from GSE94019.

Raw gene expression matrices were downloaded using the GEOquery R package (v2.66.0) and subsequently processed and quality-controlled in R software (v4.3.2; R Foundation for Statistical Computing, Vienna, Austria). To eliminate systematic bias across platforms and samples, expression values were first log2-transformed for normalization. The normalizeBetweenArrays() function was then applied for cross-sample normalization to improve comparability of expression levels between samples. Genes with more than 20% missing values or extremely low expression were removed. All data processing steps were implemented using reproducible R scripts, executed within a consistent computational environment.

Differential expression analysis

Differential expression analysis was performed using the limma R package on normalized data. The thresholds for identifying differentially expressed genes (DEGs) were set as |log2 fold change (FC)| > 1 and adjusted p-value < 0.05. The resulting DEGs were used for subsequent machine learning-based feature selection and functional analyses. Volcano plots and hierarchical clustering heatmaps were generated using ggplot2 and pheatmap, respectively, for the visualization of differential expression patterns.

Machine learning-based identification of key genes

To further identify genes most strongly associated with DR from the DEGs, two feature selection algorithms-LASSO regression and SVM-RFE-were applied.

LASSO Regression

LASSO regression was conducted using the glmnet package. By introducing an L1-regularization penalty, LASSO shrinks regression coefficients and selects the most discriminative feature genes. Ten-fold cross-validation was used to determine the optimal penalty parameter, λ, and the model with the minimum mean squared error was selected.

SVM-RFE

SVM-RFE was implemented using the e1071 package. This method iteratively removes features with the lowest importance, based on classification accuracy, thereby ranking gene importance. The gene subset corresponding to the highest classification accuracy was selected as the final SVM-RFE output.

Evaluation of diagnostic performance

The intersection of genes identified by both LASSO and SVM-RFE was defined as the set of key genes. The diagnostic ability of these genes was assessed by constructing receiver operating characteristic (ROC) curves using the pROC package. The area under the curve (AUC) was calculated, with AUC > 0.7 considered indicative of good diagnostic performance.

Functional Enrichment Analysis

Gene Ontology (GO) analysis: GO enrichment analysis of DEGs was performed using the clusterProfiler package to investigate BPs, molecular functions (MFs), and cellular components (CCs). Statistical significance was defined as p < 0.05 [8].

Kyoto Encyclopedia of Genes and Genomes (KEGG) pathway analysis: KEGG pathway enrichment was conducted using the KEGGREST and pathview packages to identify signaling pathways associated with DR-related DEGs [9].

Regulatory network prediction

To explore upstream noncoding RNA regulatory mechanisms of the key gene(s), a multi-database cross-validation strategy was employed: (i) miRNA-mRNA interactions: Candidate miRNAs potentially targeting the key gene were predicted using the miRanda, miRDB, and TargetScan databases. (ii) lncRNA-miRNA interactions: lncRNAs interacting with candidate miRNAs were predicted via the spongeScan database. Based on these results, an integrated lncRNA-miRNA-mRNA regulatory network was constructed and visualized using Cytoscape v3.9.1.

Statistical analysis

All statistical analyses were performed using R software (v4.3.2). A p-value < 0.05 was considered statistically significant.

## Results

Differential expression analysis

**Figure 1:**
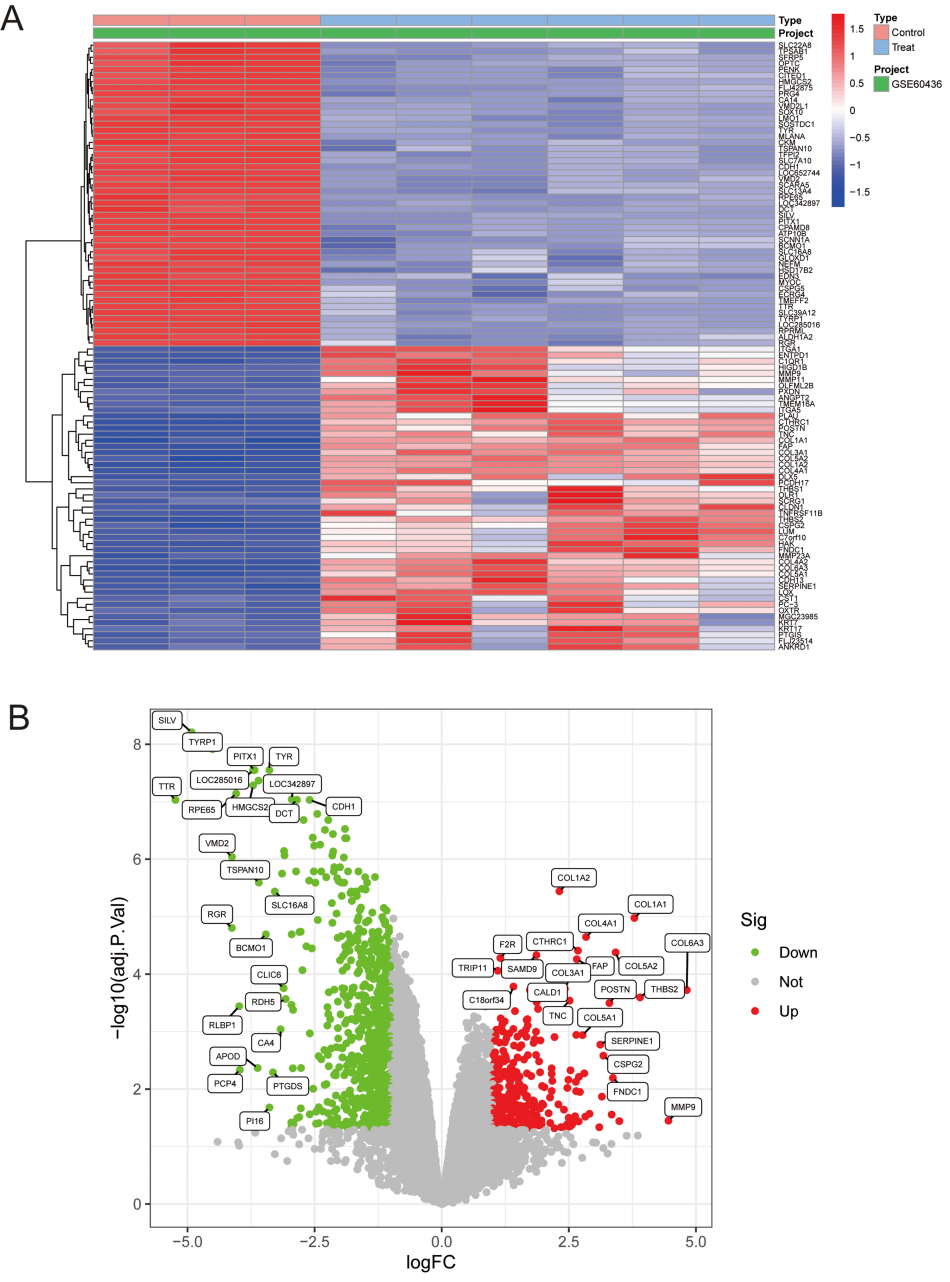
Differential expression analysis of the GSE94019 dataset (A) Heatmap of DEGs showing distinct transcriptional profiles between control and treated samples in GSE94019. Z-score-scaled expression values and hierarchical clustering highlight coordinated gene modules responsive to treatment. (B) Volcano plot displaying log2 fold changes versus adjusted p-values. Significantly upregulated (red) and downregulated (green) genes are indicated, with selected key genes labeled to illustrate major treatment-associated transcriptional shifts. DEGs, Differentially Expressed Genes

Standardization was performed for all samples using R software. A total of 790 DEGs were identified in the GSE94019 dataset, including 370 upregulated and 419 downregulated genes (Figures 1A, 1B).

Machine learning-based identification of key genes

**Figure 2:**
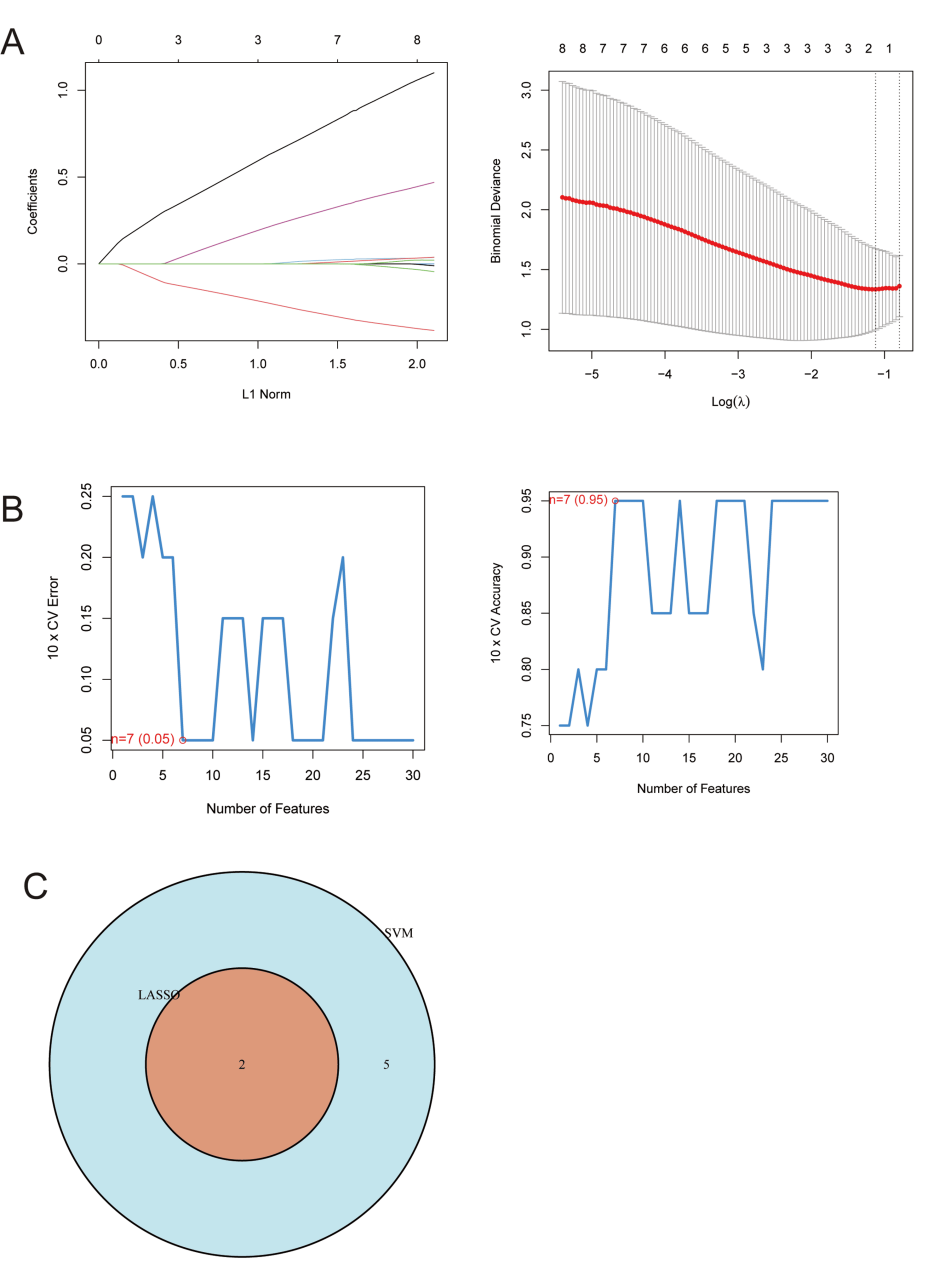
Key gene screening using two machine learning algorithms (A) LASSO coefficient profiles and λ selection: Coefficient trajectories across increasing L1 penalties (left) and 10-fold cross-validated binomial deviance across log(λ) values (right). The optimal penalization parameter was chosen using the λ_min criterion, yielding a sparse predictor set with minimal deviance. (B) SVM feature ranking and cross-validation performance: Ten-fold cross-validated classification error (left) and accuracy (right) for models incorporating increasing numbers of top-ranked features. Features were ranked using an SVM with an RBF kernel, and the optimal subset was defined as the feature count achieving maximal accuracy with minimal model expansion. (C) Intersection of selected features: Venn diagram showing overlap between LASSO- and SVM-selected predictors, with two features jointly identified across both methods. SVM, Support Vector Machine; LASSO, Least Absolute Shrinkage and Selection Operator; RBF, Radial Basis Function

To further identify genes most closely associated with DR, two complementary machine learning feature selection methods - LASSO regression and SVM-RFE - were applied. LASSO regression identified two genes, Collagen Type VI Alpha 2 Chain (COL6A2) and LINC01247 (Figure 2A). SVM-RFE analysis revealed that the highest classification accuracy was achieved at the optimal feature subset (elbow point on the x-axis in Figure 2B), yielding seven genes: COL6A2, LINC01247, ZC3H4, LOC101929199, GPX4, METRNL, and CDV3. The intersection of the two methods resulted in two overlapping genes, COL6A2 and LINC01247 (Figure 2C).

Expression levels and diagnostic performance of key genes

**Figure 3:**
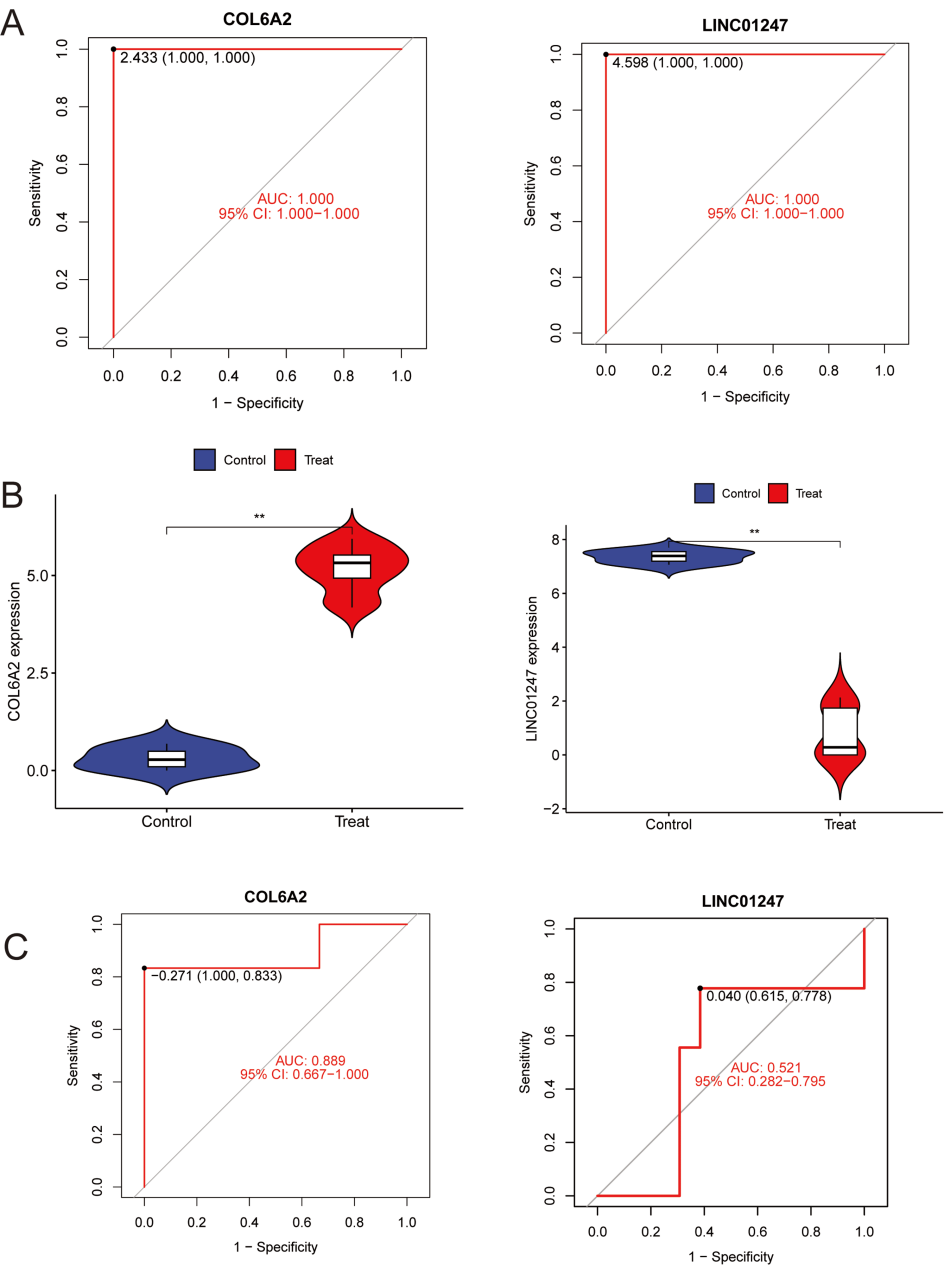
Expression levels and diagnostic performance of key genes (A) ROC curves for COL6A2 and LINC01247 in the discovery dataset: ROC analysis demonstrating near-perfect discrimination between control and treated samples for both COL6A2 and LINC01247 (AUC = 1.000; 95% CI: 1.000-1.000). Optimal cutoffs were determined using Youden’s index. (B) Gene expression differences between groups: Violin plots showing significantly elevated COL6A2 and reduced LINC01247 levels in treated samples compared with controls (p < 0.01, Wilcoxon rank-sum test). Box elements depict the median and interquartile range within each distribution. (C) External validation of diagnostic performance: ROC curves in the independent validation dataset showing strong discriminatory ability for COL6A2 (AUC = 0.889; 95% CI: 0.667-1.000) and limited performance for LINC01247 (AUC = 0.521; 95% CI: 0.282-0.795). Optimal thresholds were again computed using Youden’s index. ROC, Receiver Operating Characteristic; AUC, Area Under the Curve

To validate the expression patterns and diagnostic potential of the machine learning-identified key genes in DR, expression analyses and ROC curve evaluations were conducted using both the training dataset (GSE94019) and the validation dataset (GSE60436). Expression analysis showed that COL6A2 was significantly upregulated in DR samples compared with controls (p < 0.001), whereas LINC01247 was markedly downregulated (p < 0.001) (Figure 3A). ROC analysis revealed that the AUC for COL6A2 was approximately 1.0 in the training set (Figure 3B) and 0.89 in the validation set (Figure 3C). For LINC01247, the AUC was approximately 1.0 in the training set but only 0.521 in the validation set, indicating poor diagnostic value. Therefore, LINC01247 was excluded from subsequent analyses. The high AUC values for COL6A2 demonstrate strong sensitivity and specificity in distinguishing DR patients from healthy controls, supporting its potential as a clinically valuable molecular biomarker.

COL6A2-based differential analysis and functional enrichment

To further clarify the biological functions and molecular mechanisms of the key gene COL6A2 in DR, samples from the GSE94019 dataset were divided into high- and low-expression groups based on COL6A2 expression. Differential expression and enrichment analyses were performed accordingly.

**Figure 4:**
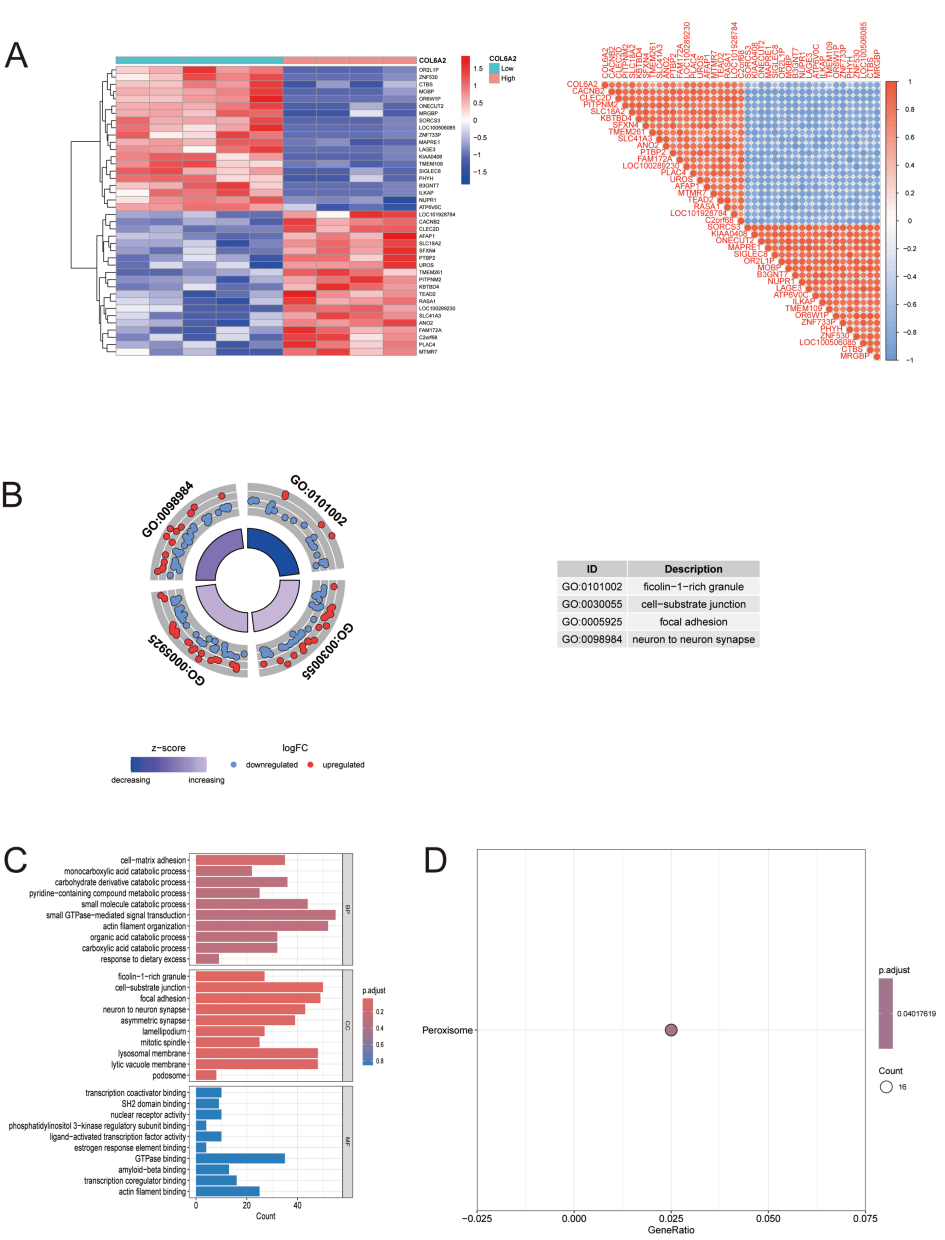
COL6A2-related differential expression and functional enrichment analyses (A) COL6A2-stratified expression heatmap and coexpression matrix. Left: Heatmap showing transcriptomic profiles of genes stratified by COL6A2 high vs. low expression, scaled by z-score and clustered by Euclidean distance. Right: Pairwise Pearson correlation matrix for COL6A2-associated genes, illustrating a coordinated high-correlation module enriched in COL6A2-high samples. (B) Gene Ontology (GO) enrichment of COL6A2-associated genes: Circular plot summarizing enriched GO terms, with outer rings indicating directionality (logFC) and inner rings indicating standardized expression (z-score). Table highlights top GO terms, including “flocculin-1–rich granule,” “cell–substrate junction,” “focal adhesion,” and “neuron to neuron synapse.” (C) Detailed GO term distribution across functional categories: Bar plots displaying GO biological process, cellular component, and molecular function enrichments for COL6A2-related genes. Bars represent enriched term frequencies; the color scale reflects adjusted p-values. (D) Kyoto Encyclopedia of Genes and Genomes (KEGG) pathway enrichment: Dot plot showing significant KEGG pathways, identifying peroxisome enrichment among COL6A2-associated transcripts. Dot size indicates gene count, and the color scale represents adjusted p-value.

A total of 1,721 DEGs were identified between the two groups, including 614 upregulated and 1,107 downregulated genes. The correlation heatmap (Figure 4A) shows that COL6A2 is positioned in the upper left region and exhibits strong positive correlations with multiple DEGs, including CACNB2, CLEC2D, PITPNM2, SLC18A2, KBTBD4, SFXN4, and TMEM261. Many of these genes are involved in ECM organization, intracellular signaling, and metabolic regulation, suggesting that COL6A2 may contribute to DR progression by cooperatively modulating extracellular microenvironment homeostasis and metabolic signaling, thereby promoting vascular pathology and tissue remodeling.

GO enrichment analysis revealed that upregulated genes were primarily enriched in ECM organization, cell adhesion, and inflammation-related functions, whereas downregulated genes were mainly associated with neuronal synaptic transmission and cellular signaling homeostasis (Figures 4B, 4C). KEGG pathway analysis identified peroxisome-related signaling as the most significantly enriched pathway (Figure 4D).

COL6A2-associated lncRNA-miRNA-mRNA regulatory network

**Figure 5:**
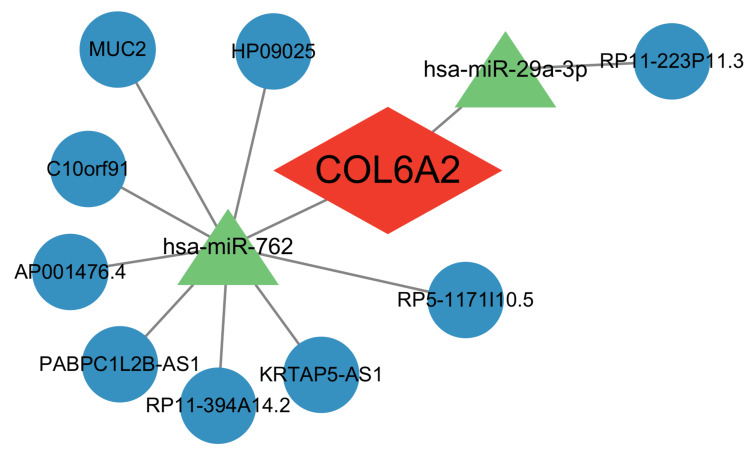
lncRNA-miRNA-mRNA regulatory network associated with COL6A2 Network visualization showing predicted regulatory interactions among COL6A2 (center, red), key miRNAs (green triangles), and their associated mRNA/lncRNA targets (blue circles). hsa-miR-762 and hsa-miR-29a-3p are identified as candidate upstream regulators of COL6A2, with each miRNA connected to its respective downstream transcript set based on shared targeting relationships. Node shapes represent RNA biotypes, and edge links denote predicted miRNA-target interactions, derived from integrated miRNA-mRNA regulatory databases.

To explore the upstream post-transcriptional regulatory mechanisms of COL6A2 in DR, potential miRNAs and lncRNAs were predicted using the miRanda, miRDB, TargetScan, and spongeScan databases. As shown in Table 1, candidate miRNAs predicted to target COL6A2 included hsa-miR-29b-3p, hsa-miR-762, hsa-miR-525-5p, hsa-miR-29a-3p, hsa-miR-29c-3p, and hsa-miR-658. According to Table 2, potential lncRNAs regulating hsa-miR-762 included HP09025, AP001476.4, MUC2, RP11-394A14.2, C10orf91, PABPC1L2B-AS1, RP5-1171I10.5, and KRTAP5-AS1. The predicted upstream lncRNA of hsa-miR-29a-3p was RP11-223P11.3. As shown in Figure 5, COL6A2 was identified as the central node in the regulatory network, with two miRNAs - hsa-miR-762 and hsa-miR-29a-3p - demonstrating the most significant regulatory associations.

**Table 1:** Predicted miRNAs potentially regulating COL6A2

Gene	miRNA	miRanda	miRDB	TargetScan	Sum
COL6A2	hsa-miR-29b-3p	1	1	1	3
COL6A2	hsa-miR-762	1	1	1	3
COL6A2	hsa-miR-525-5p	1	1	1	3
COL6A2	hsa-miR-29a-3p	1	1	1	3
COL6A2	hsa-miR-29c-3p	1	1	1	3
COL6A2	hsa-miR-658	1	1	1	3

**Table 2:** Predicted lncRNAs potentially regulating hsa-miR-762 and hsa-miR-29a-3p

miRNA	lncRNA
hsa-miR-762	HP09025
hsa-miR-762	AP001476.4
hsa-miR-762	MUC2
hsa-miR-762	RP11-394A14.2
hsa-miR-29a-3p	RP11-223P11.3
hsa-miR-762	C10orf91
hsa-miR-762	PABPC1L2B-AS1
hsa-miR-762	RP5-1171I10.5
hsa-miR-762	KRTAP5-AS1

## Discussion

DR is one of the most common microvascular complications among patients with diabetes. Its pathogenesis is multifactorial, involving hyperglycemia-induced oxidative stress, inflammatory responses, ECM remodeling, endothelial dysfunction, and neurodegenerative alterations [10]. Although current therapeutic strategies - such as laser photocoagulation, anti-VEGF agents, and vitrectomy - offer clinical benefits [11], these interventions primarily target advanced stages of the disease and are insufficient for early, precise diagnosis and molecular intervention. Therefore, elucidating the molecular mechanisms underlying DR, and identifying novel key biomarkers, is of great significance for early prevention and treatment.

In this study, we integrated bioinformatics and machine learning approaches using the publicly available DR datasets GSE94019 and GSE60436. After data normalization and differential expression analysis, LASSO regression and SVM-RFE algorithms were applied to screen for key genes, ultimately identifying COL6A2 as a candidate gene closely associated with DR. Our findings demonstrated that COL6A2 was significantly upregulated in DR samples, with ROC curve analyses revealing strong diagnostic performance (AUC > 0.85). This suggests that COL6A2 may serve as a promising molecular biomarker for early detection. COL6A2 encodes the α2 chain of type VI collagen, a major ECM structural protein involved in cell adhesion, signal transduction, and tissue stability [12]. Previous studies have shown that hyperglycemia can induce excessive collagen deposition and basement membrane thickening, leading to increased vascular permeability and microangiopathy [2]. To our knowledge, the present study is the first to report the significant upregulation of COL6A2 in DR, suggesting that it may play a broader pathological role in diabetes-related complications.

Functional enrichment analyses revealed that COL6A2-related DEGs were mainly enriched in canonical pathways, including PI3K-Akt, TGF-β, and ECM-receptor interaction signaling. These pathways are well known to participate in ECM metabolism, angiogenesis, and inflammatory regulation, indicating that COL6A2 may contribute to structural remodeling and functional impairment of the diabetic retina by modulating the ECM-integrin axis and downstream AKT activation.

Additionally, the construction of the lncRNA-miRNA-mRNA regulatory network provided insights into the upstream post-transcriptional regulation of COL6A2. The results suggested that hsa-miR-762 and hsa-miR-29a-3p may directly target COL6A2, while several lncRNAs (e.g., PABPC1L2B-AS1 and RP11-223P11.3) may regulate COL6A2 indirectly through competitive binding to these miRNAs, forming a ceRNA network. Previous research has demonstrated that the miR-29 family is downregulated in diabetic nephropathy and diabetic cardiomyopathy [13,14], where it suppresses collagen gene expression (including COL1A1, COL4A1, and COL6A2) and is closely linked to TGF-β signaling [15]. Our results are consistent with this regulatory pattern, further supporting the involvement of ceRNA-mediated modulation in DR.

Based on these findings, we hypothesize that COL6A2 may contribute to DR development through several mechanisms: (1) its upregulation increases ECM components, leading to basement membrane thickening and vascular stiffening; (2) ceRNA-mediated enhancement through lncRNA-miRNA competitive binding forms a positive feedback loop that further amplifies its expression; (3) downregulation of synaptic and neuronal signaling genes - reflected by the enrichment of the GO term “neuron-to-neuron synapse” - suggests that elevated COL6A2 may indirectly impair neuronal function, potentially contributing to early visual dysfunction and neurodegeneration observed in DR patients.

Despite these insights, this study has some limitations. First, the transcriptomic datasets were obtained from public repositories, with limited sample sizes and lacking detailed clinical staging information; thus, the findings require validation in larger, more diverse cohorts. Second, the conclusions are primarily based on computational predictions, without experimental validation. Future studies should utilize retinal endothelial cell cultures or DR animal models, combined with gene manipulation experiments, to verify the mechanistic role of COL6A2. Moreover, integrating single-cell sequencing and spatial transcriptomics could provide more precise insights into the cell type-specific expression patterns and cellular interaction networks associated with COL6A2 in the retina.

## Conclusions

In summary, this study identified COL6A2 as a key gene associated with DR using an integrated bioinformatics and machine learning approach. COL6A2 was significantly upregulated in DR tissues and was closely linked to ECM remodeling, angiogenesis, and inflammatory responses. Its expression was further modulated by multilayered noncoding RNA regulatory networks. Consistent with previous research, our results reaffirm the central role of ECM metabolic dysregulation in DR, and provide new evidence supporting COL6A2 as a potential molecular biomarker and therapeutic target. These findings offer new insights into the molecular mechanisms of DR and may contribute to precision medicine strategies for its diagnosis and treatment.
